# On the Dynamics of the Psychosocial Work Environment and Employee Well-Being: A Latent Transition Approach

**DOI:** 10.3390/ijerph18094744

**Published:** 2021-04-29

**Authors:** Ieva Urbanaviciute, Koorosh Massoudi, Cecilia Toscanelli, Hans De Witte

**Affiliations:** 1 National Centre of Competence in Research LIVES, University of Lausanne, 1015 Lausanne, Switzerland; koorosh.massoudi@unil.ch; 2 Institute of Psychology, University of Lausanne, 1015 Lausanne, Switzerland; cecilia.toscanelli@unil.ch; 3 Research Group Work, Organisational and Personnel Psychology, KU Leuven, 3000 Leuven, Belgium; hans.dewitte@kuleuven.be; 4 Optentia Research Focus Area, North-West University, Vanderbijlpark 1900, South Africa

**Keywords:** job characteristics, employee well-being, work stress, latent profiles

## Abstract

The current study investigates employee well-being in stable versus changing psychosocial working conditions, using the Job Demand-Control theoretical framework. It thereby addresses a gap in the literature dealing with how the dynamics of the work environment may affect different aspects of well-being, such as job satisfaction, work stress, mental health complaints, and overall quality of life. The study was carried out on a large heterogeneous sample of employees in Switzerland (*N* = 959) and was based on two measurement points. Latent profile and latent transition analyses were used to analyse the data. The findings revealed three commonly encountered and temporally quite stable patterns of job characteristics (i.e., latent profiles), defined by low, average, or high job control and average job demands. The average demand-low control combination was the most precarious, whereas a combination of average demands and high control was the most beneficial and it clearly outperformed the balanced average demands-average control pattern. Furthermore, our results partially supported the claim that employee well-being is contingent on the dynamics (i.e., transition scenarios) of the psychosocial work environment. They particularly highlight the central role of job resources in preventing the deleterious effects on well-being, which may occur even in relatively mild situations where job demands are not excessive.

## 1. Introduction

Psychosocial working conditions refer to important job characteristics in terms of content and work organisation [1]. They may be classified into job demands, such as heavy workload, and job resources, such as autonomy or opportunities to develop and apply one’s skills [2,3]. Major theoretical models consider them the building blocks of employees’ psychological experiences at work with lasting implications for health and well-being [4,5].

Notably, job demands and resources do not act in isolation—they are thought to interact in creating a (un)favourable work environment. This leads to an implication that different combinations of job characteristics should be considered when investigating their role in employee outcomes [5]. Moreover, adopting a temporal perspective is crucial when assessing the work environment. Being persistently versus temporarily exposed to a certain set of conditions should produce different effects on employees (see Frese & Zapf [6]), and these potentially differing effects are not yet fully understood. The majority of existing studies rely on a momentary estimation of working conditions that are then assumed to have long-term outcomes. Hence, even longitudinal investigations tend to overlook the changing nature of the work environment per se, ignoring whether a given combination of job demands and resources is persistent and how this may affect employee well-being.

In the current study, we aimed to address this gap by employing a longitudinal person-centred approach. First, we aimed to identify the most salient combinations of key job characteristics suggested in the Job Demand-Control model (i.e., job demands, decision authority (autonomy), and skill discretion) that are likely to be encountered by employees at work [1,2]. As a result, this allowed us to classify employees into latent profiles in terms of their working conditions. Second, we tested the stability and change of latent profile membership over a one-year period. Ultimately, we compared a set of health and well-being outcomes cross-sectionally across the profiles and longitudinally across the profile transition scenarios in order to investigate how exposure to a certain set of working conditions affects employee well-being.

## 2. Theoretical Background

### 2.1. Psychosocial Working Conditions

Most theoretical models define psychosocial work environment by job demands and job resources. The Job Demand-Control model [3,7] is one such model and it offers several foundational assumptions about the nature and impact of working conditions on employee well-being. It posits that job demands and job control are the key characteristics of the work environment. Job demands pertain to psychological stressors that cause strain and include aspects, such as workload, time pressure, role conflict, and the like [3,8]. Job control pertains to key job resources that help dealing with job demands. It consists of two separate dimensions referring to the possibility to use one’s skills at work and the freedom to decide how to accomplish the tasks [7,9]. One important tenet of the JDC model is that psychological job demands and job control jointly predict employee well-being depending on how they are configured together [7,10,11,12]. This suggests that a combination of demands and control rather than isolated job characteristics should be considered in order to understand how and when the psychosocial work environment affects employee outcomes. For instance, a combination of high demands and low control defines high-strain jobs and is considered conducive to ill-being (i.e., the stressor–strain hypothesis), whereas high demands accompanied by high control denote active jobs that are thought to result in positive outcomes [9,13,14]. To date, the above-mentioned assumptions have been mostly investigated focusing on additive and multiplicative effects. Additive effects refer to the main effects model, where job demand and job control variables autonomously predict employee outcomes cross-sectionally or over time. Multiplicative effects are tested in a moderation model. In this case, job control is set to buffer the effects of job demands, thereby inspecting high versus low control conditions [2,8,15]. Over the recent decades, studies testing the assumptions of the JDC model have produced abundant findings, also revealing several important shortcomings. The most notable criticism emerging from the literature is that empirical evidence about the joint impact of job demands and job control appears to be rather inconsistent. While there is quite some support for their additive effects on a range of well-being variables, the evidence about their multiplicative effects in reducing strain is much more scarce [5,10,16]. Hence, research still seems to fail to comprehensively depict the co-occurrence of job demand and control characteristics, which is a major setback in understanding their blended role in employee well-being. One reason for that may be that previous studies have either only partially managed to capture different combinations of job demands and job control or they have not aimed to do that at all. This also results in a lack of knowledge about the temporal stability of these combinations and the impact it may have on employee well-being. At this point, it may therefore be necessary to step beyond a cross-sectional and variable-centred approach in order to properly address these gaps.

### 2.2. A Person-Centred Methodological Approach to Job Characteristics

#### 2.2.1. Exploring Job Demand-Control Combinations

Studies investigating the role of job characteristics in employee well-being have for the major part used a conventional variable-centred approach. A variable-centred approach focuses on isolating characteristics on which individuals differ and then studies the correlation of these characteristics in a given sample. Hence, such analyses rely on the properties of separate variables. Whereas by adopting a person-centred approach, one gains the possibility of studying a configuration of multiple variables of interest within the person, which then becomes the centre of analysis [17]. As a result, an advantage of person-centred analytic methods, such as latent profile analysis (LPA), is that they allow for identifying unobserved subgroups of individuals based on the similarity of their scores [18], which represent qualitatively and quantitatively distinct configurations of input variables [19]. This is clearly beneficial for research on job characteristics, as it may help overcome some of its known issues and shortcomings. Notably, it helps to capture naturally emerging combinations (i.e., latent profiles) of job demands and control in the investigated sample, which is not easily done otherwise. By linking these profiles to well-being outcomes, such analyses then enable the researcher to test the combined effects of different job characteristics, thus offering a more holistic insight into the impact the work environment has on employees.

This line of investigation is gradually finding its way with quite promising results. For instance, Van den Broeck et al. [20] have distinguished four job demands and resources profiles, demonstrating that employees in demanding jobs were more at risk of high burnout and low engagement. De Spiegelaere et al. [21] succeeded in identifying five distinct job characteristics profiles in the electricity sector and showed that low-strain and active jobs were related to the best scores of work engagement and innovative work behaviour. Mäkikangas et al. [22] adopted a multi-level LPA to identify healthy and unhealthy (i.e., high-strain) departments, whereas yet another study conducted by Keller et al. [23] replicated a bipolar low stressors-high resources and low resources-high stressors profile solution across four samples and linked it to employee well-being. These and similar findings convey a very important message for further research on the topic. By pointing out the unobserved heterogeneity among employees in terms of their job characteristics, they unequivocally suggest that such heterogeneity may exist in any sample and it is crucial to unravel it in order to understand how workplace ill- and well-being evolves.

This implication has laid the foundations for the current study, in which we adopted a person-centred approach to investigate the emerging patterns of key job characteristics in the general working adult population. Drawing on the JDC model’s assumptions about different job types that are situated on a quadrant combining the job demand and job control axes [3,7], we aimed to corroborate this theoretical distinction empirically. Encouraged by the above-mentioned findings on the existence of job characteristics profiles, we thus expected to identify more than one unobserved subpopulation displaying distinct patterns of job demands and both dimensions of job control as a starting point of our study. This led to our first hypothesis:

**Hypothesis** **1** . Distinct patterns (i.e., latent profiles) of job demands, decision authority, and skill discretion should emerge denoting a differing degree of favourability of the psychosocial work environment, based on the JDC quadrant.

#### 2.2.2. Adopting a Temporal Perspective

After establishing cross-sectional combinations of job demands and job control, we subsequently aimed to extend these analyses into a longitudinal framework. Despite a steep increase in longitudinal investigations over the recent years, they often have their primary focus on the dynamics of selected outcome measures rather than job characteristics as such. For instance, some studies have examined the development of employee well-being related processes over time [24,25,26], unravelling their changes in the light of various job demands or resources. On the flipside, the dynamic nature of the work environment per se and the lasting impact it may have on the individual still needs to be better understood. Persistent exposure to (un)favourable work environment should have different implications on employee well-being compared to a temporary one (see Frese & Zapf [6]), which is a sound reason to explore these aspects more in detail. The current study thus aims to test how stability and change in the constituent characteristics of the work environment occur and what role it may play in employee outcomes. In doing so, we join rare previous attempts to address similar questions. In this regard, Igic et al. [27] have recently provided interesting evidence for the formation of different constellations of growth trends in job resources and demands over a period of 10 years. Whereas a study of Bujacz et al. [28] explored psychosocial working conditions patterns among highly skilled workers and observed some fluctuations in their prevalence over six years. To advance on the topic, we aimed to examine the longitudinal development of such patterns within a general population over a one-year period, which is long enough to capture change versus non-change in the working conditions and yet short enough to spot its imminent implications for well-being. To do so, it was necessary to identify so-called transition scenarios that denote stability and change of employee membership in the identified job characteristics profiles from one time point to another in a second step of the current study. Although some shifts are likely to occur [28], based on the build-up logic of Karasek and Theorell [7], one may also expect the emergence of pretty much stable patterns, especially given quite a short time lag. The above authors claimed that strain tends to generate further strain, which then implies that stressful (or vice versa, resourceful) job experiences may reinforce themselves, embedding the person in the same type of work environment over time. Hence, whilst we considered both types of transition patterns plausible, we expected a different degree of salience in them.

**Hypothesis** **2** . The most salient transition scenario will denote stability (i.e., staying in the same job characteristics profile), accompanied by less salient transition scenarios that denote moving from one profile to another one year later.

#### 2.2.3. Linking Well-Being Outcomes

Ultimately, we sought to unravel how the different profiles of job demands, decision authority, and skill discretion relate to employee well-being concurrently and over time. Previous person-centred research has shown some evidence that less favourable work environment types (e.g., high-strain jobs) relate to lower well-being [20,21,23]. However, such findings provide only a partial picture examining one-time effects of a given work environment or focusing on rather specific outcome indicators, such as rumination or job satisfaction [27,28]. To address this gap, we considered a broader set of balanced positive and negative aspects of employee well-being in the current study that cover both work and general life domains, are substantiated by the theory, and are suitable to be examined both as instant and as longer-term outcomes of the job characteristics profiles and transitions thereof. Job satisfaction denotes a positive emotional state resulting from the evaluation of one’s job experience [29]. It is one of the most important work-related outcomes frequently investigated within the JDC model. In the current study, we focused on global job satisfaction that refers to the evaluation of the job situation as a whole. Work stress refers to perceiving one’s work environment as taxing [30] and it is another highly relevant outcome representing a negative aspect of work-related well-being. Furthermore, the inclusion of a health indicator was substantiated by a strong emphasis on health outcomes in the JCD research that includes aspects, such as physical symptoms, subjective health, mental health, and unhealthy habits [2]. In the current study, we particularly focused on mental health that refers to anxiety and depressive symptoms among employees. The last outcome, quality of life, is a positive indicator of general well-being [31]. In the current study, it refers to an evaluation of the overall quality of one’s life at present.

In theory, favourable work environments that include high levels of job resources, such as decision authority and skill discretion, promote positive outcomes such as job satisfaction. Whereas a deterioration in employee well-being is thought to occur in unfavourable settings where job demands are not compensated by job resources, thus causing psychological strain [12,13]. Hence, we expected such effects to reflect in the levels of work stress and mental health complaints. Moreover, assuming that workplace experiences may spillover to non-work domains [32], we also expected the quality of life to be related to the favourability of the work environment that one is exposed to. Based on the above, our hypothesized instant effects are as follows:

**Hypothesis** **3.** Favourability of the work environment is concurrently linked to employee well-being: unfavourable job characteristics profiles entail lower levels of well-being (in terms of job satisfaction, work stress, mental health, and quality of life) compared to more favourable profiles at any given time point.

Besides that, drawing on Frese and Zapf [6], we expected corresponding longitudinal effects to occur. These authors have described several ways in which the stressor–strain relationships evolve over time. Remarkably, they maintain that the quality of employee functioning in the workplace (e.g., in terms of well-being) may not simply follow the presence or absence of a stressor—it may as well show accumulation effects, where ill-being increases over time due to a prolonged exposure to taxing work environment and may not instantly decline after the stressor (or unfavourable job characteristics) is removed. This served as the basis for our last set of hypotheses:

**Hypothesis** **4** . The dynamics of employee well-being over a one-year period, as expressed in the levels of job satisfaction, work stress, mental health, and quality of life, are contingent upon the job characteristics profile transition scenario.

**Hypothesis** **4a.** Stable exposure to an unfavourable work environment (i.e., staying in the same profile) results in the accumulation of ill-being, whereas stable exposure to a favourable work environment results in heightened well-being.

**Hypothesis** **4b** . Changes in the work environment (i.e., transitioning to a different profile) have asymmetrical effects on employee well-being, so that moving into a less favourable profile relates to a decrease in well-being, whereas leaving an unfavourable profile does not necessarily result in instant positive effects.

## 3. Methods

### 3.1. Procedure

The present study was based on the data obtained from a longitudinal “Professional Paths” survey conducted at the Swiss National Centre of Competence in Research—Overcoming Vulnerabilities: Life Course Perspectives (LIVES). This survey benefits from a large heterogeneous adult sample randomly drawn from the national register of inhabitants that is managed by the Swiss Federal Statistics Office (SFSO) (for more details on the sampling strategy see Maggiori et al. [33]). Participant recruitment was handled by a polling institute. The participants were invited to complete the survey by means of the invitation letter sent by post. The participation was voluntary, and the data were collected anonymously, with a six-digit code identifying each participant. At the end of each wave, participants received a compensation of 20 CHF. They could choose to either donate it to a non-profit organization or to receive a gift card in this amount.

### 3.2. Sample

#### 3.2.1. Sample Characteristics

The data from two waves collected in 2016 and 2017 were used in the present study, with a one-year lag between the measurements. The final sample consisted of 959 employed adults (50.6% female; mean age at T1 = 46.67, *SD* = 8.21), reflecting the German- and French-speaking Swiss population in terms of age, gender, and linguistic region. With regard to education, 37% of participants held a higher education degree (*n* = 358), 47% had an upper secondary or vocational education diploma, or its equivalent (*n* = 448), 3% had compulsory education (*n* = 31), and the remaining sample indicated other type of education or did not respond to this question. Approximately 95% of the participants were employed on a permanent basis in their main job (*n* = 917 at T1 and *n* = 914 at T2). The household income, measured as an ordinal variable, ranged from (1) less than 40,000 CHF to (8) over 160,000 CHF per year.

#### 3.2.2. Selection Criteria

To be included in the sample, the participants had to have participated in both waves of the study and have at least partially responded on the variables of interest. Furthermore, a criterion of being professionally active was applied when composing the final sample. Only data from participants who held a remunerated employment contract during both measurement occasions were included in the analyses. Holding the same job at both time points, however, was not a prerequisite. The majority of participants (*n* = 886) stayed with the same employer, whereas a small fraction (*n* = 73) changed their job.

#### 3.2.3. Sample Attrition

At Time 1, the initial valid sample consisted of 1172 employed adults. At Time 2, the valid sample decreased to 959 employed adults. Some participants were eliminated because they became unemployed or professionally inactive (*n* = 40), the rest dropped out from the study (*n* = 173). We compared the dropout and the final sample and found no significant differences in participants’ age, gender, or type of contract. However, the dropout sample reported lower household income, Δ*M* = 0.45, *p* = 0.010 and showed a different distribution in the level of education, χ^2^(2) = 6.56, *p* = 0.038, containing a higher percentage of less educated participants than the main sample. With regard to psychological variables, no differences were detected, except for lower quality of life among the dropouts as measured at Time 1, Δ*M* = 0.20, *p* = 0.001.

### 3.3. Measures

Job characteristics were assessed with 14 items from the Job Content Questionnaire (JCQ) [34]. The items were rated on a four-point scale (1 = strongly disagree, to 4 = strongly agree) and were subdivided into three subscales measuring decision authority (3 items, Cronbach’s α_T1_ = 0.82, α_T2_ = 0.83), skill discretion (6 items, Cronbach’s α_T1_ = 0.75, α_T2_ = 0.72), and psychological demands (5 items, Cronbach’s α_T1_ = 0.61, α_T2_ = 0.61). Whilst the latter falls in the lowest acceptable range of reliability [35], it is comparable to a number of studies that showed similar psychometric properties of this subscale [36,37,38].

To evaluate job satisfaction, a one-item measure was used. It was developed for the aims of the Professional Paths survey and asked the participants to evaluate the overall satisfaction with their current job using a four-point response scale (1 = not satisfied at all, to 4 = very satisfied).

Work stress was assessed with the General Work Stress Scale (GWSS) [30]. It consists of nine items measuring subjectively experienced work stress (e.g., “Does your work make you so stressed that you wish you had a different job?”). The responses were recorded on a 5-point scale (1 = never to 4 = always). Cronbach’s α_T1_ = 0.90, α_T2_ = 0.91.

Quality of life was measured with a one-item scale. The participants were asked to rate their health overall quality of life on a five-point response scale (1 = very bad to 5 = very good).

Mental health complaints were measured with a six-item subscale from the General Health Questionnaire (GHQ-12) [39]. The participants had to rate depression and anxiety symptoms they had experienced recently (e.g., “Have you recently lost sleep over worry?”). The items had to be rated on a four-point response scale (1 = not at all, to 4 = much more than usual), Cronbach’s α_T1_ = 0.87, α_T2_ = 0.88.

Because this is a two-wave study, multi-item scales were checked for longitudinal invariance and satisfied the requirement of metric invariance across the two time points.

### 3.4. Data Analyses

#### 3.4.1. Latent Profiles and Latent Profile Transition

The analyses were conducted in three steps. First, separate series of LPA [40] were performed to examine unobserved subgroups of employees with regard to their job characteristics at Time 1 and Time 2. This was done as a prerequisite for the longitudinal LPA and latent transition analyses conducted in steps 2 and 3 and allowed for determining the optimal latent profile solution at each time point. The mean scores from the JCQ subscales of decision authority, skill discretion, and psychological demands were used as indicators for the LPA. In each series, we took the one-profile model as a baseline, increasing the number of profiles until the optimal solution was reached, as per guidelines in the literature [41]. Decision about which profile solution should be retained was based on their fit statistics as well as on the interpretability of profiles. The following fit statistics were inspected: the Akaike Information Criterion (AIC), the Bayesian Information Criterion (BIC), the sample-adjusted BIC (SaBIC), Lo–Mendell–Rubin likelihood ratio test (LMR), the Bootstrap Likelihood Ratio Test (BLRT), and entropy. Lower values of the AIC, BIC and SABIC indicate a better fitting model. A non-significant value of the LMR and BLRT tests, obtained after comparing a *k*-profile model with a *k*-1 profile model, indicates that a more parsimonious model should be kept. Entropy informs about the classification accuracy, values closer to one indicating a better solution.

In step two, we selected the most optimal set of latent profiles obtained at each time point and estimated them simultaneously without modelling a transition yet. In this step, we applied a sequential procedure aimed at testing the equivalence of Time 1 and Time 2 profile solutions with regard to their means and variances. To this end, we gradually imposed model constraints starting with an unconstrained model, then constraining the means in the corresponding profiles across the two time points, and ultimately adding variance constraints.

In step three, a latent transition analysis (LTA) [41,42] model was tested based on the best fitting model from step two. It is a longitudinal extension of latent profile analyses, which allows for the investigation of latent profile membership stability and change over time by regressing Time 2 profiles on Time 1 profiles. To account for occasional missing data, full-information maximum likelihood (FIML) estimation was used in both LPA and LTA.

#### 3.4.2. Covariates and Outcomes

Participants’ background characteristics were modelled as covariates of latent profile membership in LPA using the auxiliary variable command. We used the R3STEP command in Mplus (for more details see [43]), allowing to directly examine the covariates without imposing bias to the profile solution [44]. Furthermore, cross-sectional outcome analyses were conducted using the BCH command [44,45]. It yields a comparison of the mean levels of outcomes across the job characteristics profiles identified in LPA.

Finally, longitudinal analyses were carried out based on profile transition scenarios. To this end, transition scores from the LTA were saved to a data file. Based on these scores, a change in employee well-being outcomes in each latent profile transition scenario was inspected using repeated measure analyses with two time points. To test the role of employer change in profile transitions, we combined their scenarios into two groups, the first group encompassing all employees who transitioned to a different profile and the second group encompassing all employees with stability scenarios. Then, a Chi-Square test of independence was used to inspect the frequency of job changers across these two groups.

## 4. Results

Descriptive statistics are provided in Table 1 and inform about the means, standard deviations and inter-correlations of the main study variables at both time points. Additionally, Table A1 in the Appendix A displays correlations between background variables and latent profile indicators (i.e., job characteristics).

### 4.1. LPA Results

The results of job characteristics latent profile analyses conducted in the first step are summarized in the upper part of Table 2 and provide a comparison of the alternative latent profile solutions at Time 1 and Time 2.

**Figure A1:**
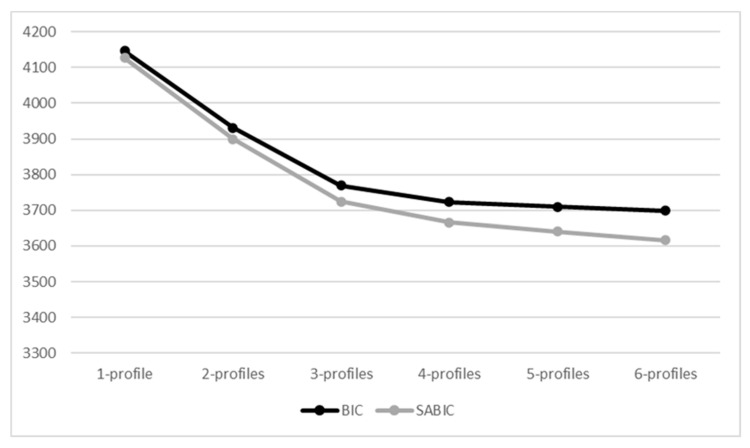
BIC and SABIC plots based on Time 1 LPA.

**Figure A2:**
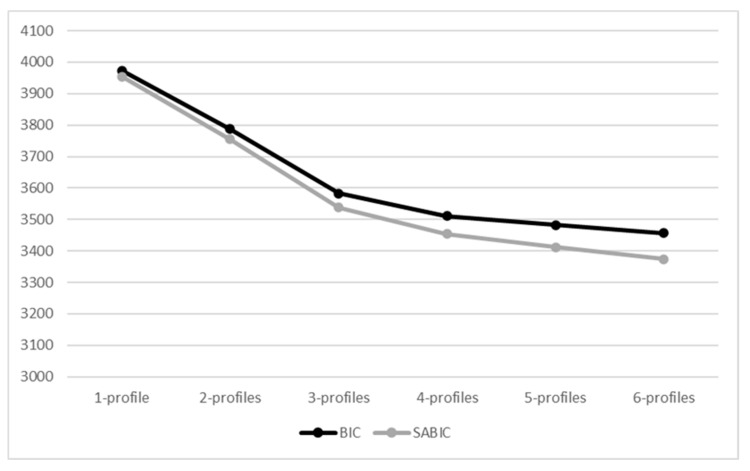
BIC and SABIC plots based on Time 2 LPA.

As seen in Table 2, the three-, four-, and five-profile solutions had quite good fit statistics at both time points. The LMR and BLRT tests were significant in all cases. However, the most notable decrease in the information criteria, such as BIC and SABIC, was observed when switching from two to three profiles at Time 1 as well as Time 2 (see Figure A1 and Figure A2 in the Appendix A), suggesting a three-profile solution. Furthermore, despite that the four- and five-profile solutions (and a six-profile solution at Time 2) had slightly higher entropy than the three-profile solution, they contained a negligible profile at both time points that was not well interpretable. Based on these results, as well as on the interpretability of the profiles, the three-profile solution was chosen as the optimal one.

To inspect the comparability of the profiles, in the next step we gradually increased the constraints, beginning with a configural model with freely estimated means and variances across the two time points, proceeding to a model where profile means were constrained to equality over time, and ultimately, to a model where both means and variances were set to be equal over time. Mean equality refers to so-called structural similarity, whereas constraining the variances helps to establish the similarity of dispersion, thus increasing the comparability of Time 1 and Time 2 latent profile solutions [46]. The results are provided in the lower section of Table 2 and show that the models did not differ considerably with regard to the information criteria and entropy. Hence, the most parsimonious three-profile model with constrained means and variances was retained for further analyses.

**Figure 1:**
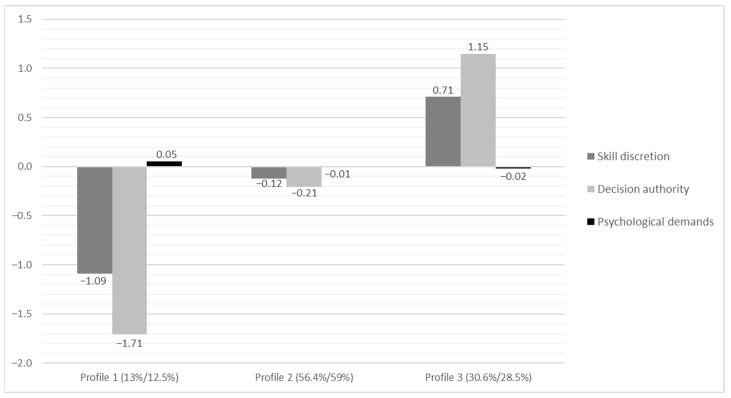
The final three-profile solution after imposing invariance constraints over time in LTA. For easier interpretation, the graph is based on z scores. Profile 1 = Low resources profile. Profile 2 = Average profile. Profile 3 = High resources profile. Percentages before the slash indicate the size of the profiles at Time 1. Percentages after the slash indicate the size of the profiles at Time 2.

As shown in Figure 1, we labelled the first profile the “low resources” profile. It characterizes employees with low decision authority (i.e., autonomy), low opportunities for skill utilization, and average psychological job demands. The “average” profile refers to employees with average scores on all three job characteristics, whereas the “high resources” profile consists of employees who reported high decision authority and skill discretion. Covariate analyses showed that age and contract type did not predict profile membership. Regarding gender, women had lower odds of being classified into the high resources profile (at Time 2 only) as compared with the low resources profile. Other advantageous background characteristics, such as higher level of education and higher household income, were associated with higher odds of being classified into a more favourable profile (see Table A2 and Table A3 in the Appendix A for more details).

### 4.2. LTA Results

A latent transition analysis was run based on the above-described constrained three-profile solution. In the current study, the LTA had nine possible transition scenarios. Detailed information on transition probabilities and final counts for each scenario is provided in the Appendix A (see Table A4 and Table A5). According to the results, profile membership proved to be quite stable over time at ~80% rate and we found no evidence that employer change would play a role: the proportion of job changers was similar among those who stayed in the same profile and those who transitioned to a different one over time, χ^2^(1) = 0.001, *p* = 0.980. Three salient job characteristics stability scenarios were identified, denoting stable low resources (*n* = 92), stable average (*n* = 463), and stable high resources (*n* = 219) scenarios. Concerning latent profile change, four transition scenarios were retained for further analyses, denoting the average-to-high (*n* = 50), high-to-average (*n* = 79), low-to-average (*n* = 22), and average-to-low (*n* = 26) transitions. Extreme transition scenarios (i.e., moving between the high and low resources profile) were very scarce and they were excluded from outcome analyses for this reason.

### 4.3. Outcome Analyses Results

Cross-sectional results are provided in Table 3 and show the mean levels of employee well-being across the latent profiles at any given time point.

Almost all pairwise-comparisons showed significant mean differences. The high resources profile was associated with the highest scores in positive well-being indicators (i.e., job satisfaction and quality of life) and the lowest scores on ill-being (i.e., work stress and mental health complaints). By way of contrast, employees with the most precarious low resources profile reported the lowest scores of well-being as compared with more favourable average or high resources profiles at both time points.

**Figure 2:**
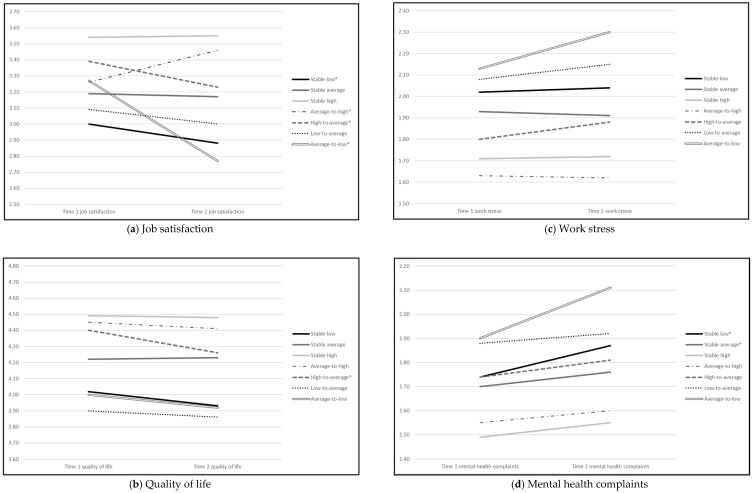
Change in employee well-being across profile transition scenarios. Asterisks in the legend indicate a significant change in a given aspect of well-being from Time 1 to Time 2 in the marked transition scenarios.

The results of repeated measure analyses are provided in Figure 2 and they inform about the change in employee well-being over time due to the transition scenario. Looking from a temporal perspective, our findings suggest that staying in a favourable profile was related to persistently higher levels of well-being but not to accumulation of it, as seen in the case of the stable-high-resources scenario. Some accumulation of ill-being may be implied in the stable-low-resources scenario, as we observed a significant decrease in job satisfaction and a significant increase in mental health complaints, even though the latter was slightly increasing in the entire sample, across all transition scenarios. Moreover, transitioning to a different profile was only related to changes in positive but not negative indicators of well-being. Job satisfaction was the most malleable outcome, with a significant decrease in its levels as employees transitioned to a less favourable profile (i.e., high-to-average and average-to-low transition scenarios) and an increase in its levels in the average-to-high transition scenario. The low-to-average transition did not result in a significant improvement of job satisfaction and even showed a slight decline. A significant decrease in the levels of quality of life, another positive outcome, was mostly associated with the high-to-average transition scenario. Other profile change scenarios, however, were not related to a corresponding change in this outcome over time.

## 5. Discussion

### 5.1. Interpretation of the Job Characteristics Profiles

The current study provides an insight into the ways vulnerability and flourishing at work take place by unravelling the dynamic relationship between the work environment and employee well-being. In line with Hypothesis 1, the findings revealed three patterns of job characteristics characterized by high, average, and low job control resources and average job demands. This indicates sample heterogeneity with regard to their typical working conditions and could be expected both from a theoretical and from an empirical point of view. The JDC model adopts a typological approach towards the work environment, defining it by different combinations of job demands and job control. Our findings corroborate this approach in the sense that we did observe varying levels of key job characteristics—job control resources in particular—across the identified profiles. The observed set of profiles, however, did not exactly match the four job types discussed in the JDC research [3,9,14], which may be due to the fact that some types of jobs are less prevalent in the world of work and may not always emerge [8]. Such mismatch seems to be quite common: previous person-centred studies have identified anywhere from two [23] to five [21] work environment profiles, and these variations are natural because latent profiles always reflect the characteristics and experiences of a specific sample.

Notably, whilst we used a large and rather heterogeneous sample, descriptive statistics revealed the overall quality of their working conditions to be higher than average. This means that the emergence of a large enough sub-group with extreme vulnerabilities (i.e., high strain) was less likely among our participants. Such around-average trends shown not only here but can be also found in large-scale European data. For example, the sixth European Working Conditions Survey indicated the Swiss work intensity index to be slightly below and skills and discretion index to be very close to the overall European average [47]. This lends a useful explanation for the shape of the profiles obtained in the current study—they all had average levels of job demands and were mostly differentiated based on the levels of decision authority (i.e., autonomy) and skill discretion. In other words, we did not observe the typical “high-strain” and “active” combinations with a highly expressed demands dimension but rather see a milder variant of them in the low resources and high resources profiles correspondingly, whereas the largest “average resources” profile seems to represent the above-mentioned Swiss standard with both job demands and job control expressed around the midpoint. 

As expected in Hypothesis 2, the majority of participants remained in the same working conditions over the studied period, with similar probability rates for staying in the most and least favourable profiles. This adds to existing findings [28] and is in line with theoretical assumptions suggesting that job types have an underlying dynamic that promotes the continuity of a given job pattern [7]. Stated otherwise, for someone in a high-strain job (or low resources job in our case), a lack of resources may not allow for adequately meeting the job demands, which will further reinforce the resource–demand imbalance, thereby establishing a strain pattern. A similar rationale applies to so-called active jobs (or high resources jobs in our case): resourceful employees get more activated, which fosters their job resources and increases the probability of maintaining a favourable working conditions pattern over time. Such reasoning closely approximates the principles of conservation of resources [48], which maintain that resource dynamics are inherent in the stress experience. Resource depletion and elevated levels of stress are reciprocally interlinked, enclosing people in a loss cycle, which explains why they cannot easily switch from an unfavourable to a more favourable pattern. Whereas in the case of resource availability, the opposite dynamic should be promoted, making it easier to maintain favourable conditions over time.

At this point, it is notable that job change was unrelated to transitioning to a different working conditions pattern. However, background variables such as male gender, higher level of education, and higher initial financial status were all found to increase one’s chances of having a more favourable type of job, such results implying that socio-economic status may play a role in determining the quality of one’s job and, in a way, the quality of employment in general.

### 5.2. Interpretation of Findings on Well-Being Outcomes

The main contribution of the current study is that it unravels the impact that different patterns of job characteristics may have on employee outcomes. Our results have largely supported Hypothesis 3 showing that, from a cross-sectional perspective, the more resourceful the work environment, the more it relates to higher employee well-being with obvious differences across the profiles (see Table 3 for the summary of findings). The high resources job characteristics profile is particularly distinguishable as it was associated with significantly higher well-being on all aspects, at both times points, and as compared to both the low resources and the adjacent average resources profile. In turn, the low resources pattern showed stark differences from the opposite-end high resources pattern and, in most cases, from the average resources pattern. These findings, first of all, serve as a sound validation of the three-profile solution as such, showing that the profiles discriminate well between the outcomes. Second, they hint at the importance of increasing access to job resources, since such remarkable differences in employee well-being across the profiles seem to be due to variations in the job control dimension. Third, they suggest that even average job demands may create a precarious work environment if the resources are not sufficient.

Our longitudinal results bring more light to such considerations, addressing the call for more research on the temporal dynamics of stressor–strain reactions at work [6,14,49,50]. As summarized in Figure 2, most changes were found in job satisfaction, which provides an illustrative case of how degrading versus improving working conditions may trigger a corresponding change in well-being. Such findings can be thought to reflect the first phase of several exposure time models encompassing an increased initial reaction to the stressor. Although Frese and Zapf’s work [6] mostly concentrates on stressor–strain reactions, the current study provides some evidence of the reversed dynamic as well, linking resource increase to flourishing at work. Remarkably, in addition to the changing scenarios, our results also showed a decline in job satisfaction in the stable and most unfavourable “low resources” scenario, but we did not observe a corresponding accumulation of well-being in the favourably stable scenarios. It is an intriguing finding that conforms to Hypothesis 4 and suggests that positive and negative effects may be not symmetrical. From a theoretical point of view, it falls in line with Karasek and Theorell’s [7] reasoning that strain creates more strain, thus even stable but unfavourable working conditions can result in a degrading well-being.

Such tendencies, however, do not apply universally to all investigated outcomes. Quality of life showed to be less malleable, which may be attributable to the nature of the construct. Compared to job satisfaction, which denotes an immediate reaction to existing psychosocial working conditions, it represents a more distal and static outcome covering multiple areas, not just work [31]. Therefore, fluctuations in job control may have been not strong enough to cause significant changes in the overall quality of life or the time lag may have been too short to observe them.

Furthermore, the aspects of ill-being either were not subject to change (i.e., work stress) or their change seemed to make part of an overall growth trend observed within the population (i.e., mental health complaints). While somewhat unexpected, one explanation for such findings lies in the contents of our identified profiles. Notably, they varied in the levels of job control resources but not demands, and this variation in positive job characteristics possibly targets positive aspects of well-being in the first place. Whereas according to the JDC logic [3,8], one would expect strain reactions to occur due to an increase in job demands, which remained virtually the same across different scenarios in our case. It is, however, important to note that degrading working conditions (i.e., average-to-low transition scenario) found some resonance in both aspects of ill-being, but this trend did not reach the significance level, likely due to the tiny fraction of the sample (*n* = 26) that was exposed to it.

### 5.3. Implications and Limitations

The current study gives an additional vantage point for discussing the makeup of an optimal work environment. While in theory the most beneficial active job profile is conceptualized by the combination of high resources and high demands, already Karasek and Theorell [7] have noted that demand levels should be high but not excessive. Some empirical studies have even concluded that low-demands and high-control jobs may produce more desirable effects than high-demands and high-control jobs [50,51], thus launching a debate in the literature about which combination is the most favourable. In this context, our “high resources” profile is particularly intriguing as it may indicate a perfect job demands–resources match, and the current findings on elevated well-being associated with it seem to point in that direction.

This further touches upon the role of job resources versus job demands in employee well-being. A lot of attention within the JCD literature has been given to high psychological demands that are inherent in high-strain jobs. While there is no doubt about the deleterious consequences of such work environments [16,21,52], they represent quite an extreme end. To elaborate on the very same inquiry, it may be crucial to examine various intermediate variants as they can inform about which job characteristics are indispensable for separating a favourable work environment from an unfavourable one. Given the rather schematic (i.e., high vs. low) approach towards the job characteristics, such questions have rarely been tested in the JDC literature. We had a unique opportunity to do it here, and our study adds to the existing literature by showing that even milder variants of these theoretical job types can account for substantial differences in well-being.

From a practical point of view, the current findings have demonstrated that the psychosocial work environment can be perceived as quite dynamic and it immediately affects employee outcomes, especially job satisfaction. The fact that it can either deteriorate or improve over quite short periods of time, even when staying with the same employer, indicates the importance of preventive and reactive HR interventions in keeping the right balance between demanding and resourceful job characteristics on a regular basis. Our analyses have clearly shown that even a slight difference in job resources may matter much. It is remarkable that the average resources profile, which seems to be the most common in the population and overall is quite well-balanced, still does not produce sustainable well-being and was found to be significantly less optimal than the high resources profile. This particularly encourages investing in various job resources in organizations and teaching employees how to capitalize on them. In today’s turbulent world of work, job demands that are determined by external labour market factors may be difficult to adjust or remove, whereas the advantage of psychosocial job resources is that they are often at organization’s and supervisor’s disposal and this can help make a difference in the way a work environment is experienced and affects employees’ well-being.

As in every study, our findings are not exempt from limitations that are important to note and address in future investigations. First, we consider it essential to expand and upgrade the measurement of psychosocial job characteristics. In the current study, the psychological job demands subscale performed quite poorly and it may have been one of the reasons why we did not observe much variation in job demands across the identified profiles. While using a well-known measure increases the comparability of findings, a few concerns have been raised in the literature with regard to inconsistent reliability of its scores [37], as well as a lack of precision of the construct [53,54]. Future research should focus on these aspects to better capture the variety of job demands. It would be particularly useful to separate between hindrance and challenge demands as they are known to have a different impact on employee outcomes [55]. Additionally, one may be interested in expanding the list of characteristics that are pertinent in today’s organizations (e.g., management regimes, level of perceived responsibility, work/time arrangements, specialised skills) and consider integrating objective indicators among them, as self-report measures portray the reality from a subjective perspective only.

Second, although the current study identified several vulnerability scenarios (e.g., transition from average to low resources), they were encountered by a rather small proportion of the sample, which complicates their comparison with substantially larger non-vulnerable groups. Dropout analyses have also shown that the dropouts had somewhat lower quality of life compared to the remaining sample. This means that vulnerable participants tended to quit the study, lowering the chances of identifying big enough groups of employees with a vulnerability profile. Future studies may specifically address this issue by using targeted sampling procedures. This would allow for a better insight into the way vulnerabilities evolve among the most fragile members of the working population.

Third, there is room for advancement in the investigation of stability and change in the work environment. Whilst the present study revealed several interesting scenarios, we do not know the pre-history of the pattern observed at Time 1, that is, for how long the person had been exposed to it. This drawback may explain why our results on changes in well-being were quite inconsistent. To circumvent this issue and to further inspect the stressor–strain models delineated by Frese and Zapf [6], future studies may consider using samples where change has an a priori set starting point, such as newcomers [50] or those whose organizations undergo a stressful period. It would be as well pertinent to focus on longer time lags as, for example, in Igic et al. [27]. Such investigations would offer an opportunity to explore the “entrapment” patterns from a career development perspective that are of particular importance in turbulent times.

Finally, since the current study was focused on well-being outcomes, we only tested standard background characteristics as covariates of the job characteristics patterns encountered by our participants. A logical next step would be to go beyond the socio-demographic predictors by including personal and psychosocial context variables as they may better reveal personal and structural resources that help people escape precarious settings and have more satisfying working lives.

## 6. Conclusions

The current study has identified three patterns of job characteristics denoting salient low, average, and high job control resources and average job demands. While such combinations do not fully correspond to the job types described in the JDC model, they can be considered milder variants of them. According to the findings, people tended to stay embedded in their job type over time, irrespective of whether they had changed employer or not, only one fifth of the sample transitioning from one pattern to another. Cross-sectional comparisons clearly demonstrated the high resources pattern to be the most beneficial, whereas the low resources pattern showed detrimental effects. Longitudinal findings were less consistent, but they also suggest that an average demands-high resources (but not average resources) work environment may be the one leading to sustainable well-being. In contrast, both deteriorating working conditions and prolonged exposure to a resource-deprived work environment showed harmful signs, especially touching upon job satisfaction. Such findings connect the dynamics of the work environment to the dynamics of well-being. Their key message is that vulnerability scenarios at work may be determined by the level of job control resources solely, and they seem to occur in relatively mild situations where job demands are not necessarily excessive.

## Figures and Tables

**Table 1:** Descriptive statistics and correlations between the main study variables.

Variables	M (*SD*)	1	2	3	4	5	6	7	8	9	10	11	12	13
1. T1 JCD-skill	3.04 (0.46)													
2. T1 JCD-auto	3.10 (0.61)	0.54 ***												
3. T1 JCD-dem	2.61 (0.44)	0.18 ***	−0.05											
4. T1 Jobsat	3.27 (0.58)	0.26 ***	0.31 ***	−0.25 ***										
5. T1 Wstress	1.87 (0.62)	−0.02	−0.17 ***	0.41 ***	−0.47 ***									
6. T1 QL	4.28 (0.65)	0.20 ***	0.26 ***	−0.11 **	0.24 ***	−0.31 ***								
7. T1 MH	1.66 (0.57)	−0.08 *	−0.14 ***	0.26 ***	−0.32 ***	0.61 ***	−0.40 ***							
8. T2 JCD-skill	3.05 (0.44)	0.75 ***	0.43 ***	0.17 ***	0.19 ***	−0.02	0.21 ***	−0.09 **						
9. T2 JCD-auto	3.10 (0.60)	0.44 ***	0.67 ***	−0.04	0.22 ***	−0.19 ***	0.23 ***	−0.16 ***	0.53 ***					
10. T2 JCD-dem	2.61 (0.42)	0.17 ***	−0.02	0.62 ***	−0.17 ***	0.31 ***	−0.10 **	0.22 ***	0.19 ***	−0.04				
11. T2 Jobsat	3.24 (0.59)	0.21 ***	0.29 ***	−0.19 ***	0.48 ***	−0.35 ***	0.24 ***	−0.29 ***	0.29 ***	0.38 ***	−0.23 ***			
12. T2 Wstress	1.87 (0.64)	<0.01	−0.15 ***	0.32 ***	−0.31 ***	0.69 ***	−0.27 ***	0.49 ***	−0.03	−0.23 ***	0.40 ***	−0.49 ***		
13. T2 QL	4.25 (0.68)	0.21 ***	0.24 ***	−0.06	0.22 ***	−0.26 ***	0.61 ***	−0.32 ***	0.25 ***	0.25 ***	−0.13 ***	0.35 ***	−0.36 ***	
14. T2 MH	1.73 (0.64)	−0.09 **	−0.14 ***	0.16 ***	−0.23 ***	0.42 ***	−0.29 ***	0.56 ***	−0.13 ***	−0.21 ***	0.23 ***	−0.39 ***	0.59 ***	−0.45 ***

Note. T1 = Time 1. T2 = Time 2. JCD-skill = skill discretion. JCD-auto = decision authority. JDC-dem = psychological demands. Jobsat = job satisfaction. Wstress = work stress. QL = quality of life. MH = mental health complaints. *** *p* < 0.001, ** *p* < 0.01, * *p* < 0.05

**Table 2:** Latent profile solutions and their fit statistics.

Model Estimation Steps	AIC	BIC	SaBIC	LMR (*p*)	BLRT (*p*)	Entropy	Smallest Profile (%)
*LPA Time 1*							
1-profile solution	4117.309	4146.504	4127.448	-	-	1.000	100
2-profile solution	3882.880	3931.539	3899.779	0.007	<0.001	0.570	34.9
3-profile solution	3700.847	3768.969	3724.506	<0.001	<0.001	0.847	11.3
4-profile solution	3635.454	3723.040	3665.873	0.031	<0.001	0.901	1.8
5-profile solution	3603.149	3710.199	3640.328	0.039	<0.001	0.802	2.0
6-profile solution	3572.382	3698.895	3616.320	0.162	<0.001	0.802	1.0
*LPA Time 2*							
1-profile solution	3944.652	3973.847	3954.792	-	-	1.000	100
2-profile solution	3738.577	3787.236	3755.476	0.007	<0.001	0.509	43.2
3-profile solution	3514.577	3582.699	3538.235	<0.001	<0.001	0.882	12.5
4-profile solution	3423.185	3510.771	3453.604	<0.001	<0.001	0.932	1.2
5-profile solution	3374.967	3482.017	3412.146	<0.001	<0.001	0.930	1.0
6-profile solution	3330.157	3456.670	3374.095	0.007	<0.001	0.940	0.9
*LPA Time 1−Time 2 tests of equivalence*							
3-3 profile model unconstrained	7215.424	7351.669	7262.741	-	-	0.864	11.3–12.5
3-3 profile model means constrained	7208.188	7300.640	7240.297	-	-	0.863	12.3–11.9
3-3 profile model means and variances constrained	7205.127	7282.981	7232.166	-	-	0.863	12.4–11.8
*LTA Time 1 -> Time 2*							
3->3 model means and variances constrained	6741.817	6839.135	6775.616	-	-	0.865	13.0–12.5

Note. LMR and BLRT are not available in single profile models and models with two time points.

**Table 3:** Cross-sectional differences in employee well-being across the job characteristics profiles.

	Job Characteristics Profiles			
Well-Being Indicators	Low Resources	Average Resources	High Resources	Overall Test
T1 Job satisfaction	2.95	3.19	3.52	88.98 ***
T2 Job satisfaction	2.82	3.17	3.58	153.46 ***
T1 Work stress	2.10	1.91	1.71	30.26 ***
T2 Work stress	2.13	1.91	1.67	42.10 ***
T1 Mental health complaints	1.82 ^n^	1.70 ^n^	1.54	18.13 ***
T2 Mental health complaints	1.96	1.78	1.54	37.87 ***
T1 Quality of life	3.90	4.25	4.47	48.87 ***
T2 Quality of life	3.84	4.24	4.45	49.23 ***

Note. The overall test assesses the overall between-profile differences (*** *p* < 0.001). It is based on a Chi-Square test with 2 degrees of freedom. All pairwise between-profile differences are significant (*p* < 0.05), except for the difference in mental health complaints between the low and average resources profiles, marked with “n”.

## Data Availability

The archiving of the Professional Paths survey data is currently in progress. The 2016 and 2017 datasets that were used in the current study are archived at FORSBASE repository https://forsbase.unil.ch/ (accessed on 31 March 2021).

## Appendix A

**Table A1:** Correlations between demographic variables and latent profile indicators.

Variables	T1 Latent Profile Indicators			T2 Latent Profile Indicators		
	JCD-Skill	JCD-Auto	JCD-Dem	JCD-Skill	JCD-Auto	JCD-Dem
Age	0.04	0.08 *	−0.05	0.05	0.06	−0.05
Gender	−0.04	−0.08 **	−0.06	−0.05	−0.07 *	−0.04
Education	0.36 ***	0.19 ***	0.08 *	0.34 ***	0.20 ***	0.09 *
T1 Contract type	−0.02	0.06	0.02	0.01	0.06	0.08 *
T1 Household income	0.25 ***	0.19 ***	0.11 **	0.25 ***	0.14 ***	0.10 **
T2 Contract type	−0.01	0.05	−0.03	0.01	0.06	0.07 *
T2 Household income	0.28 ***	0.18 ***	0.12 ***	0.28 ***	0.15 ***	0.10 **

**Notes.** T1 = Time 1. T2 = Time 2. JCD-skill = skill discretion. JCD-auto = decision authority. JDC-dem = psychological demands. Gender: 0 = male; 1 = female. Contract type: 0 = temporary; 1 = permanent. Education and household income measured in an increasing order. *** *p* < 0.001, ** *p* < 0.01, * *p* < 0.05.

**Table A2:** Covariates of the latent profile membership at Time 1.

Covariates	Compared Profiles	Odds Ratio	95%CI
Age	ns	ns	ns
Gender (female)	ns	ns	ns
Education (high)	2 vs. 1	2.55	[1.646;3.963]
	3 vs. 1	4.44	[2.726;7.238]
	3 vs. 2	1.74	[1.243;2.434]
Contract type (permanent)	ns	ns	ns
Household income (high)	2 vs. 1	1.39	[1.213;1.587]
	3 vs. 1	1.50	[1.305;1.730]

Notes. Profile 1 = low resources; Profile 2 = average resources; Profile 3 = high resources. ns = no significant results observed. Gender: 1 = male; 2 = female. Contract type: 1 = temporary; 2 = permanent. Education and household income measured in an increasing order. Only significant results indicating higher odds (i.e., OR > 1) of a given covariate in one profile versus another are shown, when the 95%CI do not include 1.

**Table A3:** Covariates of the latent profile membership at Time 2.

Covariates	Compared Profiles	Odds Ratio	95% CI
Age	ns	ns	ns
Gender (female)	1 vs. 3	1.70	[1.059;2.713]
Education (high)	2 vs. 1	1.78	[1.187;2.662]
	3 vs. 1	3.67	[2.347;5.734]
	3 vs. 2	2.06	[1.499;2.840]
Contract type (permanent)	ns	ns	ns
Household income (high)	2 vs. 1	1.17	[1.053;1.299]
	3 vs. 1	1.30	[1.160;1.462]
	3 vs. 2	1.11	[1.025;1.209]

Notes. Profile 1 = low resources; Profile 2 = average resources; Profile 3 = high resources. ns = no significant results observed. Gender: 1 = male; 2 = female. Contract type: 1 = temporary; 2 = permanent. Education and household income measured in an increasing order. Only significant results indicating higher odds (i.e., OR > 1) of a given covariate in one profile versus another are shown, when the 95% CI do not include 1.

**Table A4:** Transition probabilities.

Time 2: Time 1:	Profile 1	Profile 2	Profile 3
Profile 1	0.736	0.223	0.041
Profile 2	0.048	0.860	0.092
Profile 3	0.006	0.249	0.745

Note. Profile 1 = Low resources. Profile 2 = Average. Profile 3 = High resources.

**Table A5:** Final counts for each profile transition scenario.

Time 2: Time 1:	Profile 1	Profile 2	Profile 3
Profile 1	92	22	6
Profile 2	26	463	50
Profile 3	2	79	219

Note. Profile 1 = Low resources. Profile 2 = Average. Profile 3 = High resources.
